# New Insights into the Reactivity of Detonation Nanodiamonds during the First Stages of Graphitization

**DOI:** 10.3390/nano11102671

**Published:** 2021-10-11

**Authors:** Florent Ducrozet, Hugues A. Girard, Jocelyne Leroy, Eric Larquet, Ileana Florea, Emilie Brun, Cécile Sicard-Roselli, Jean-Charles Arnault

**Affiliations:** 1 Université Paris-Saclay, CEA, CNRS, NIMBE, CEDEX, 91191 Gif sur Yvette, France; florent.ducrozet@cea.fr (F.D.); jocelyne.leroy@cea.fr (J.L.); 2 Institut de Chimie Physique, UMR 8000, CNRS, Université Paris-Saclay, 91405 Orsay, France; emilie.brun@universite-paris-saclay.fr (E.B.); cecile.sicard@universite-paris-saclay.fr (C.S.-R.); 3 Condensed Matter Physics Laboratory (PMC), UMR CNRS 7643, Ecole Polytechnique, IP-Paris, 91228 Palaiseau, France; eric.larquet@polytechnique.edu; 4 Laboratory of Physics of Interfaces and Thin Films (LPICM), UMR CNRS 7647, Ecole Polytechnique, IP-Paris, 91228 Palaiseau, France; lenuta-ileana.florea@polytechnique.edu

**Keywords:** nanodiamonds, surface graphitization, X-ray photoelectron spectroscopy

## Abstract

The present study aims to compare the early stages of graphitization of the same DND source for two annealing atmospheres (primary vacuum, argon at atmospheric pressure) in an identical set-up. DND samples are finely characterized by a combination of complementary techniques (FTIR, Raman, XPS, HR-TEM) to highlight the induced modifications for temperature up to 1100 °C. The annealing atmosphere has a significant impact on the graphitization kinetics with a higher fraction of sp^2^-C formed under vacuum compared to argon for the same temperature. Whatever the annealing atmosphere, carbon hydrogen bonds are created at the DND surface during annealing according to FTIR. A “nano effect”, specific to the <10 nm size of DND, exalts the extreme surface chemistry in XPS analysis. According to HR-TEM images, the graphitization is limited to the first outer shell even for DND annealed at 1100 °C under vacuum.

## 1. Introduction

Since the last decade, diamond nanoparticle or nanodiamond (ND) has been the object of a growing interest from researchers, engineers, and companies. Indeed, ND gather the outstanding physical and chemical properties of the bulk diamond and new assets conferred by the nanoscale. Among the different ND sources, nanodiamonds produced by detonation (DND) are actively investigated, mainly for bioapplications, catalysis, novel lubricants, and composites [1,2,3,4]. For instance, DND are widely used as vectors for delivery of drugs or genetic material [5,6] for their <10 nm diameter provides a high specific surface area (up to 400 m^2^/g) and allows to expect renal clearance [7]. Their carbon-related surface chemistry plays a central role as it greatly influences the surface charge of DND for an efficient electrostatic loading of biomolecules of interest [8], and it strongly drives their colloidal properties governing interactions with solvents [9]. Apart from its biomedical applications, this versatile surface chemistry also offers optimized chemical bonding possibilities with oils or polymer matrix toward novel lubricants and composites materials, thereby maximizing the expected mechanical, electrical, or thermal properties. Furthermore, surface chemistry also drives DND surface-related properties such as (photo)catalytic abilities [10,11], radiosensitization [12,13], and radicals overproduction [14] or antibacterial behavior [15].

For all these applications, if oxidized and hydrogenated surfaces have already been widely investigated, graphitized surfaces may also be promising and yet intricate. As shown by Lin et al. [16], the graphitization state of DND strongly drives their catalytic behavior, as the sp^2^/sp^3^ interface may offer favorable electron transfer routes if carefully designed. The formation of a sp^2^-organized surface covering an sp^3^ core may have promising properties conferred by fullerene or graphene assets such as photothermal therapy [17]. Optimized functionalization routes were also developed on graphitized DND, via arylation reactions, for instance, to graft complex organic moieties for bioapplications or polymer composites [18].

The formation of fully graphitized DND up to onion-like carbon (OLC) structures from DND is now well documented. Indeed, as OLC have promising applications for energy storage, catalysis, and composites [19,20], the formation of OLC from ND has been extensively investigated [21]. This approach allows the creation of high-purity material and small sizes at low cost. The full transformation of ND into OLC structures was thus studied experimentally by annealing at temperatures included between 1100 °C and 1900 °C under different atmospheres (vacuum [22], argon [23], helium [24], nitrogen [25], hydrogen [26]). In parallel, formations of curved graphite-like structures and concentric-shells fullerenes on nanodiamonds were theoretically investigated [27,28,29]. In this context, a few years ago, Zeiger et al. published a complete review on OLC synthesis and energy storage applications that includes the current state of the art on graphitization mechanisms of ND [21]. According to previous investigations, thermal effects induce first the desorption of water molecules and then one of the different oxygen functional groups present at ND surface (hydroxyl, ether, carbonyl, carboxyl, anhydride, lactone). Consequently, dangling bonds are created on sp^3^-carbon atoms from the DND surface that can either be saturated by species or molecules present in the annealing atmosphere or combined together to form sp^2^ carbon local bonds. The surface graphitization seems to also be initiated from the non-diamond carbon present at the ND surface within the 700–800 °C temperature range. The reorganization of the complete first outer shell of ND as a carbon onion shell occurs for higher annealing temperatures, typically in the 900–1100 °C range. However, the primary steps of sp^3^-C to sp^2^-C transition at the DND’s surface remain less understood and finally only few studies have really focused on the first stages of graphitization under vacuum [18,30,31,32,33,34]. Furthermore, from the best of our knowledge, in such studies, the impact of the annealing atmosphere on the early stages of graphitization mechanisms of DND was not investigated in detail.

The present study aims to compare the early stages of graphitization of the same DND source for two different annealing atmospheres (primary vacuum and argon at atmospheric pressure) in an identical set-up. All DND samples are finely characterized by a combination of complementary techniques to highlight the induced modifications for temperature up to 1100 °C. FTIR and XPS investigations allow probing the effects on the surface chemistry, whereas Raman and HR-TEM observations evidence the evolution of the carbon hybridization and of the crystalline structure versus the annealing temperature for each atmosphere. The sensitivity of each technique to sp^2^ carbon is compared. We show that the annealing atmosphere has an impact on the graphitization kinetics. Whatever the annealing atmosphere, carbon hydrogen bonds are created at the DND surface during annealing according to FTIR, and the origin of the involved hydrogen species is discussed. Furthermore, we also evidence a “nano effect” on XPS analysis which exalts the extreme surface chemistry, specific to the <10 nm size of DND.

## 2. Materials and Methods

### 2.1. Nanodiamond Powder

Detonation nanodiamonds (DND) were purchased from Adamas Nanotechnologies (ND5nmN, carboxylated, Raleigh, NC, USA). This initial particle will be referred here as “initial DND”. TEM observations allowed the measurement of the Feret diameter considering 170 particles; it was centered around 6 nm.

### 2.2. Annealing under Vacuum

Annealing treatments of DND were realized in a tubular furnace of Carbolite Gero company (Sheffield, UK). A quartz tube was filled with 60 mg of initial ND in the center and inserted in the middle of the furnace. The tube was connected to a primary pumping (≈10^−3^ mbar). Vacuum was achieved and maintained for 10 min before any annealing treatment to favor the desorption of water and impurities. Annealing was then performed at temperatures from 800 to 1100 °C for 4 h while maintaining pumping. The temperature ramp for heating was maintained around 70 °C/min. 

### 2.3. Annealing under Argon

Annealing under argon atmosphere (99.9997% purity) was realized with the same equipment at atmospheric pressure. The tube was connected to argon with a 20 SCCM flux. The tube was first heated to 300 °C for 20 min to allow the desorption of water and impurities to avoid a pre-pumping step. Annealing was then performed at temperatures from 800 to 950 °C for 4 h. Temperatures were controlled with a type K thermocouple introduced in the furnace. The measured accuracy on expected temperatures was ±1.5 °C. The temperature ramp for the heating periods was maintained at around 70 °C/min.

### 2.4. Homogenization of Annealed DND in Water

To allow their analysis in a reproducible manner, the DND were homogenized in water just after annealing. Annealed powders were collected in a 15 mL plastic tube and 3 mL of ultrapure water (18.2 MΩ·cm) were added. Homogenization of the powder was obtained by a sonication step of 30 min with a 1 s on/off period and an amplitude of 60% (Cup Horn Bioblock Scientific 750 W, equipped with a cooling system, Illkirch-Graffenstaden, France).

### 2.5. Fourier Transform Infrared (FTIR) Measurements

Infrared spectra were recorded with a Bruker Vertex 70 spectrometer equipped with a diamond ATR system (Billerica, MA, USA). A measure of 2 μL of DND in water were deposited and dried on the ATR crystal (MIRacle, PIKE Technologies, Fitchburg, WI, USA) before analysis. Acquisition represents the average of 64 scans recorded with a 4 cm^−1^ resolution at room temperature and ambient atmosphere with a nitrogen-cooled MCT detector. A break was applied in the 2280–2400 cm^−1^ region to avoid the contribution of atmospheric CO_2_ absorption bands. A baseline correction was applied for analyses of DND annealed at 900 and 950 °C under vacuum.

### 2.6. X-ray Photoelectron Spectroscopy (XPS) Analysis

A measure of 10 μL of DND in water was deposited on a silicon substrate covered by a gold coating made by evaporation to limit the charge phenomenon during analysis. The substrates were dried and then analyzed. XPS measurements were performed on a Kratos Analytical Axis Ultra DLD spectrometer equipped with a monochromated Al Kα (1486.6 eV) X-ray source and a charge compensation system (Manchester, UK). The take-off angle was set at 90° relative to the sample surface. Spectra were acquired at a pass energy of 160 eV for the survey, 40 eV for core levels (O 1s, N 1s, C 1s, Zr 3d). C 1s spectra shown in this study were acquired at 10 eV to reach a higher energy resolution. Binding energies were referenced to the Au4f_7/2_ peak located at 84 eV. After the background subtraction by a Shirley correction, a curve fitting procedure was carried out to extract the components of the C1s core level using Voigt functions with a Lorentzian to Gaussian ratio of 30%. For the component of sp^2^ carbon, an asymmetry factor was added.

### 2.7. Raman Analysis

Raman spectra were recorded with a Horiba Xplora spectrometer equipped with a 532 nm laser with a 0.79 mW power (Kyoto, Japan). 10 μL of DND in water were deposited on a silicon substrate and dried. Each spectrum is the average of 3 acquisitions realized at different positions on the substrate. The acquisition time is one minute, cumulated 10 times.

### 2.8. Thermogravimetric Analysis (TGA)

TGA measurements under vacuum and argon atmosphere were realized with a Netzsch STA 449 C (Selb, Germany). An alumina crucible filled with about 3 mg of initial particles was used for each analysis. Analysis was performed from 23 to 1100 °C with a 20 °C/min heating and a 20 mL/min argon flux.

### 2.9. High-Resolution Transmission Electron Microscopy (HR-TEM) Observations

Transmission electron microscopy (TEM) was performed on a Thermo Fisher Scientific™ G3 Titan Themis 300 transmission electron microscope (C-Twin objective lens: Cs = 2.7 mm, Cc = 2.7 mm, Focal length = 3.5 mm, Waltham, MA, USA) operating at 300 kV accelerating voltage. Prior to the observation, the DND were deposited on a 3 mm diameter copper grid covered with a holey carbon film. To analyze the impact of the annealing conditions on the morphological and structural characteristics of the samples, HR-TEM observations were performed at 520,000× magnification for different defocus values using low dose mode on a Falcon3 EC 4k/4k Direct Detection Electron (DDE) camera. In order to best preserve samples from electron beam irradiation during image acquisition, a total electron dose of 25 e^−^/Å^2^ was used for a limited exposure time of 1 s.

## 3. Results

**Figure 1:**
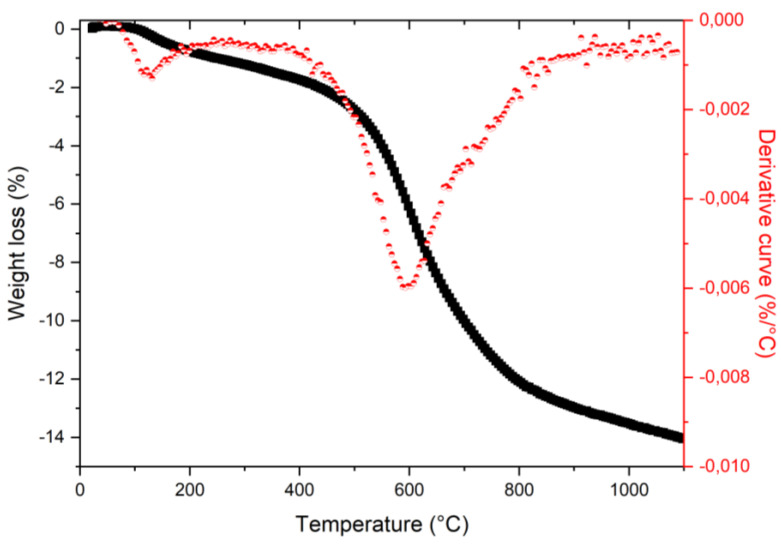
TGA spectra of initial DND annealed under argon atmosphere (black) and TGA derivative curve (red).

Thermogravimetric analysis (TGA) was first performed on initial DND under argon atmosphere at atmospheric pressure to determine the desorption thresholds of adsorbed water and oxygen functional groups (Figure 1). As expected, two main mass drops can be observed, well evidenced by the derivative function. The first one, which corresponds to the release of free, loosely, and tightly bound water [35], occurs between 100 °C and 200 °C, while the second mass drop, located between 500 °C and 700 °C, corresponds to carbon-oxygen function removal at DND surface [35]. Beyond 800°C, the decrease of the desorption rate points out that most of the carbon-oxygen functions should have been removed. This temperature then appears a pertinent one for starting our annealing thermal range.

DND were then annealed in a tubular furnace during 4 h at 800, 850, 900 and 950 °C, under vacuum or argon atmospheres to monitor the surface modifications. Annealed samples were resuspended in water and a sonication step was realized to break and separate the biggest agglomerates. Each sample was then characterized by FTIR, XPS, Raman, and HR-TEM to probe and compare their inner and surface chemistry, their carbon hybridization, and their crystallographic structure and morphology.

**Figure 2:**
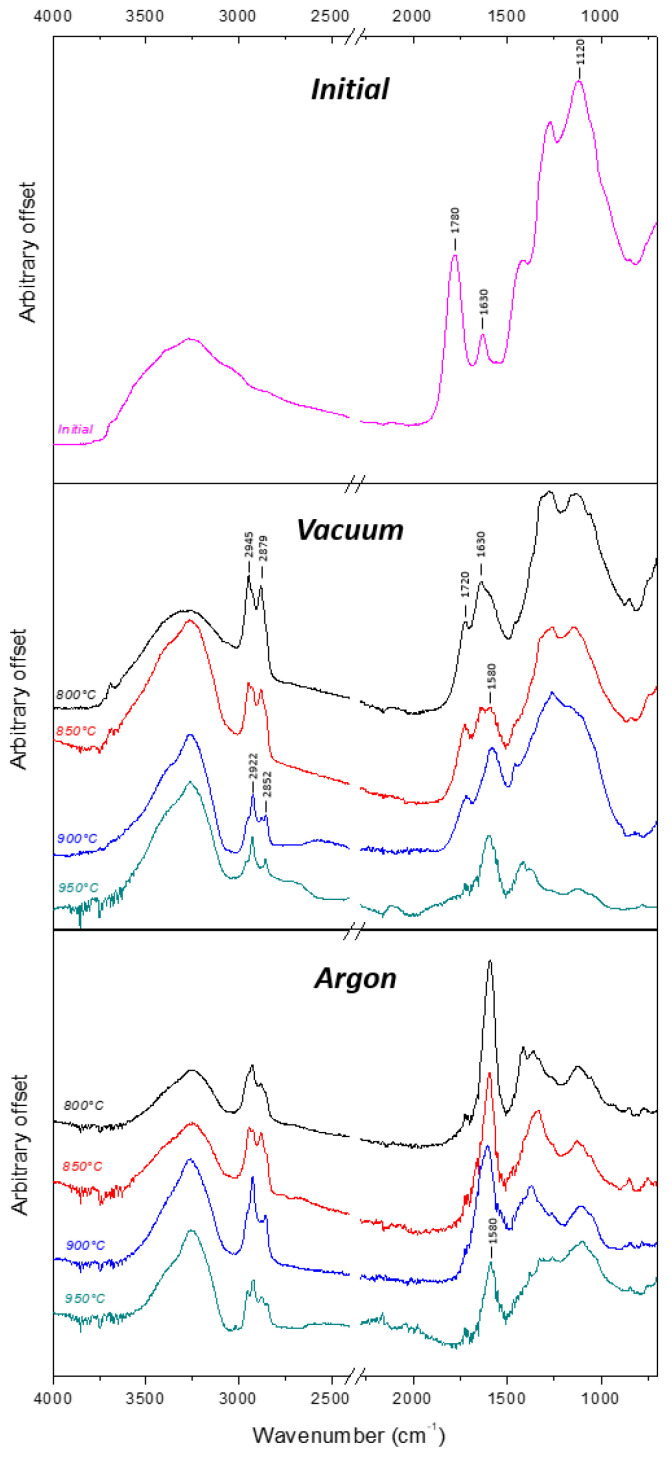
FTIR spectra of initial and annealed DND from 800 °C to 950 °C for both atmospheres.

The evolution of FTIR spectra from the initial DND to the annealed samples is depicted on Figure 2. For all samples, bands located at 3250 cm^−1^ and 1630 cm^−1^ due to O–H stretching and O–H scissor bending modes may be partially or totally related to the presence of water molecules since ATR measurements were performed under air at ambient temperature.

The spectrum of the initial DND is characterized by a prominent C=O carboxylic stretching band at 1780 cm^−1^ accompanied with another pronounced band around 1120 cm^−1^ related to single bounded carbon-oxygen groups such as etheric or alcoholic functions. This is in agreement with the specifications given by the DND manufacturer (ADAMAS), i.e., oxidized surface. The peak at 1630 cm^−1^ is assigned to O–H scissor bending frequency, which corresponds to hydroxyl groups involved in the carboxylic functions and atmospheric water. It can be noticed that there is no clear visible sign of C–H stretching modes around 2800–2900 cm^−1^ [36].

For DND annealed under vacuum, the C=O stretching band was red-shifted to 1720 cm^−1^, in closer agreement with the ketones and aldehydes wavenumber range [36] associated with a progressive reduction of its area along with the raise of the annealing temperature. At the same time, alcoholic and etheric related bands around 1120 cm^−1^ are still present, with reduced intensities, and vanish at 950 °C. Annealing under vacuum also brings interesting new features on the FTIR spectra. Already present at 800 °C as a shoulder, a new band appeared around 1580 cm^−1^ and is clearly visible at 900 °C. This band was previously assigned to C=C bonds from benzene structures, which appeared after vacuum annealing realized at 750 °C [37,38]. Nevertheless, another assignment was proposed by Petit et al. and Stehlik et al., who linked this band to red-shifted OH bending modes related to specific water interaction with the hydrophobic surface of the hydrogenated DND [38,39]. The origin of this band will be further discussed. For all samples annealed under vacuum, we also noticed an intensity increase of C–H stretching modes. At 800 °C and 850 °C, these bands are located at 2945 and 2879 cm^−1^. Surprisingly, these C–H stretching modes exactly correspond to C–H signatures of DND voluntary hydrogenated either by annealing or plasma treatments under H_2_, as reported in literature [40]. However, for annealing temperatures higher than 900 °C, these bands underwent a red-shift to 2922 and 2852 cm^−1^.

For samples annealed under argon atmosphere, a similar evolution versus the temperature can be seen with a strong reduction of C=O and C–O bands up to an almost complete removal of C=O stretching band at 1720 cm^−1^. Here as well, C–H stretching bands appear from 800 °C (Figure 2), with a more balanced ratio between CH_3_ (2945 and 2879 cm−1) and CH_2_ (2922 and 2852 cm^−1^) asymmetric and symmetric stretching modes from 800 °C to 950 °C. For these particles, the band at 1580 cm^−1^ becomes prominent from 800 °C. This is a significant difference compared to vacuum atmosphere.

**Figure 3:**
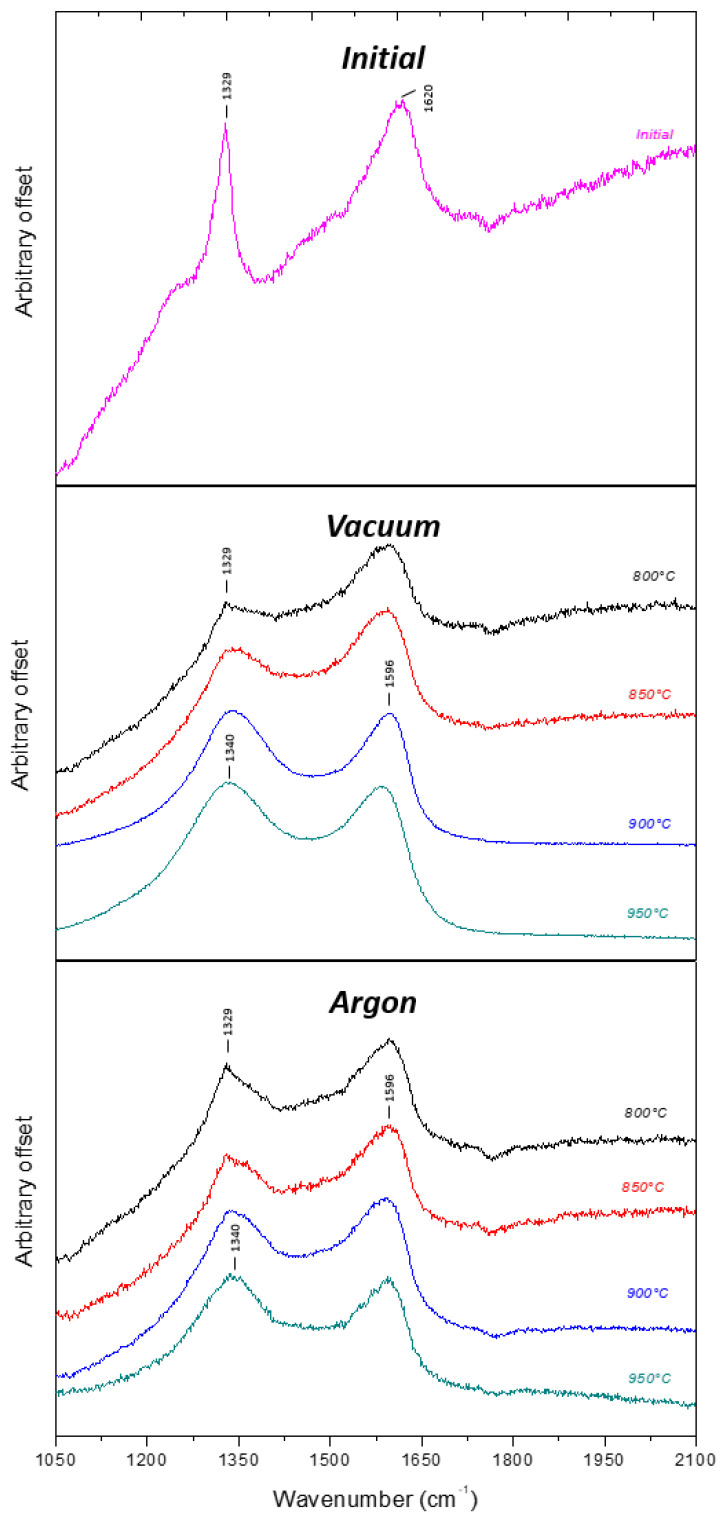
Raman spectra of initial and annealed DND from 800 °C to 950 °C for both atmospheres. The excitation wavelength was 532 nm.

Modifications of carbon hybridization induced by DND annealing were then probed by Raman spectroscopy (Figure 3).

On the spectrum of initial DND, the first order peak of diamond, corresponding to the DND’ core, lies at 1329 cm^−1^. It is red-shifted compared to bulk diamond due to the nanometric size of DND [41,42]. A broad peak centered around 1610 cm^−1^, usually named G-band, is in fact the superposition of at least three components (sp^2^ carbon 1590 cm^−1^, OH- bending 1640 cm^−1^, C=O- stretching 1740 cm^−1^) [40,42]. This G band is exalted in visible Raman analysis due to the intense scattering of sp^2^ bonded carbons compared to sp^3^ ones, by a factor of 50 [43]. On these initial DND, the D band corresponding to disordered carbon expected at 1340 cm^−1^ looks weak. The shape of the baseline also evidences a noticeable photoluminescence background arising from the sample. For vacuum, at the first annealing temperature, the diamond peak is still distinguishable but becomes no more visible for higher temperature treatment, being concealed in the D band at 1340 cm^−1^ that corresponds to disordered carbon. The contribution of oxidized functions is lowered in aid of the G band that can clearly be seen with a maximum at 1596 cm^−1^. Under argon atmosphere, a similar evolution can be observed versus annealing temperature. The diamond peak remains detectable at 1329 cm^−1^ up to 850 °C. To conclude, for both atmospheres, beyond 850 °C, Raman spectroscopy performed with a green excitation reveals that a signature of a disordered carbon material has completely replaced the initial one. At the same time, we also noticed a vanishing of the photoluminescence background on these annealed samples.

**Figure 4:**
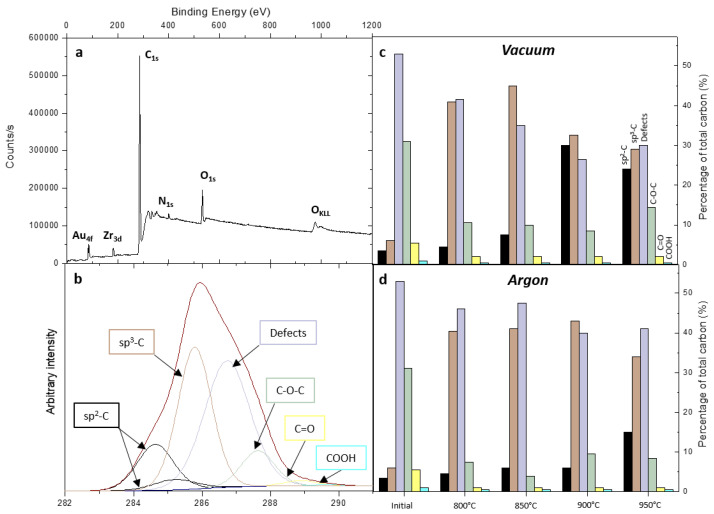
(**a**) Wide XPS spectrum; (**b**) typical C1s core level spectrum with fitting: DND after annealing at 950 °C under argon atmosphere; evolution of the six different components at C1s for initial and DND annealed under (**c**) vacuum and (**d**) argon atmospheres.

To investigate the annealing consequences on atomic concentrations of each element present in the DND and on the carbon binding states, XPS analysis was performed on initial and annealed DND. A typical wide spectrum is shown on Figure 4a. The 4f peaks of gold originate from the substrate (see experimental part). In addition to photoemission peaks of carbon, nitrogen, and oxygen, the XPS analysis revealed the presence of zirconium. The corresponding atomic concentration is between 0.6 and 0.9 at. %. The binding energy of Zr 3d doublet corresponds to zirconium oxide [44]. Its origin is probably related to the deagglomeration milling process applied to DND that used zirconium oxide beads [45]. Therefore, the oxygen atomic concentrations provided in the following were corrected from the contribution of zirconium oxide assuming a stoichiometric zirconium oxide (ZrO_2_).

For the initial DND, the oxygen and nitrogen atomic concentrations were 9.3 and 1.9 at. %, respectively (Table 1). Oxygen arises from the different functional groups initially present at DND surface and from remaining adsorbed water, as shown by FTIR (Figure 2). The main nitrogen contribution measured by XPS is related to nitrogen atoms incorporated into the DND core during the detonation synthesis [46]. This is in agreement with literature where nitrogen concentrations up to 6 at. % were measured by XPS for DND of different origins [47].

For DND annealed under vacuum, the oxygen concentration drops from 9.3 to 5.7 at. % after the first annealing at 800°C. This appears to be in agreement with the TGA analysis (Figure 1) and FTIR spectroscopy (Figure 2). Then, the oxygen concentration remains almost stable for higher annealing temperatures (Table 1). This will be further discussed. For nitrogen, a decrease from 1.9 to 1.3 at. % is obtained. Similar trends are measured for DND annealed under argon atmosphere, especially for nitrogen. Moreover, an even lower oxygen atomic concentration seems to be reached at high temperatures (3.6–4.0 at. %).

In order to gain further insights into the carbon chemistry of annealed DND, systematic deconvolution of the C1s core levels of each sample was performed. A typical C1s core level spectrum is presented on Figure 4b. Other fitted C1s core levels are provided in Supplementary Materials (Figure S1). It corresponds to DND annealed at 950°C under argon. After a background Shirley correction, different components have been considered to fit this spectrum. The sp^3^ carbon one at 285.4 eV was taken as reference, reflecting intrinsic diamond. The component related to sp^2^ carbon is downshifted at −1 eV [48]. Its asymmetry, linked to its conductor character, was taken into account in the fit (Figure 4b). With an upshift of +1 eV from sp^3^ carbon, a third component is assigned to take into account all carbon atoms not linked to an oxygen atom. This includes sp^3^ C–C neighboring structural defects commonly present in detonation ND, carbon partially saturated with hydrogen (at the surface and in the core of the particle) and carbon bounded to a nitrogen atom [31,48]. Components assigned to ether (C–O–C), C=O, and carboxyl (COOH) bonds are also present at higher binding energies, located, respectively, at +1.9, +3 and +4 eV from sp^3^ carbon [49]. All C1s spectra were fitted using these six components. Percentages of total carbon for each component are reported on Figure 4c,d for both annealing atmospheres. On initial DND, the component related to defective sp^3^ C–C, CH_x_ and C–N bonds represents 53% of the total carbon at C1s. Components linked to carbon-oxygen bonds are also preeminent, all together at 37%. The sp^3^-C contribution is weak (6%) while a small sp^2^-C is detected (3.5%).

Whatever the annealing atmosphere, the C1s spectrum undergoes strong modifications after the first annealing at 800 °C. Indeed, the contributions of C–O–C, C=O, and COOH bonds drop from 37% to 9% (argon) and 13% (vacuum) of total carbon. Considering the stoichiometry of the different carbon-oxygen groups, this decrease is in good agreement with the decrease of the oxygen atomic concentration (Table 1) and FTIR observations, considering that adsorbed water may also participate to the oxygen content. The sp^3^-C contribution arises (40–41%) and the component assigned to defects, CH_x_ and C–N bonds is reduced (42% and 46% for vacuum and argon, respectively) though a slight increase of sp^2^-C is measured (4.5%). A reorganization of the C–C bonds seems to occur, which is more likely due to surface desorption of carbon-oxygen functions but can also be explained by a migration of vacancies contained in the diamond core up to the surface, as it is well known in the field of NV centers synthesis for such temperature [50].

For vacuum annealing, the sp^2^-C part is increasing to 7.5% at 850°C, whereas it rises to 30% at 900 °C. Regarding the part of defects, CH_x_ and C–N bonds is further reduced to 35% and 26%, respectively. The sp^3^-C contribution represents the major part of carbon at 45% for 850 °C while it decreases to 32% at 900 °C. At higher temperatures, the sp^2^-C and sp^3^-C are, respectively, 24 and 29%. The component assigned to defects, CH_x_ and C–N bonds is 30%.

Looking at C1s spectra, DND annealed under argon atmosphere underwent less modification for comparable temperatures. More specifically, a different trend for sp^2^-C versus temperature is observed. Indeed, this contribution remains low at 6% below 900 °C. The part of sp^2^-C finally rises to 15% at 950 °C. The part of defects, CH_x_ and C–N bonds decreases from 48% to 40–41%.

**Figure 5:**
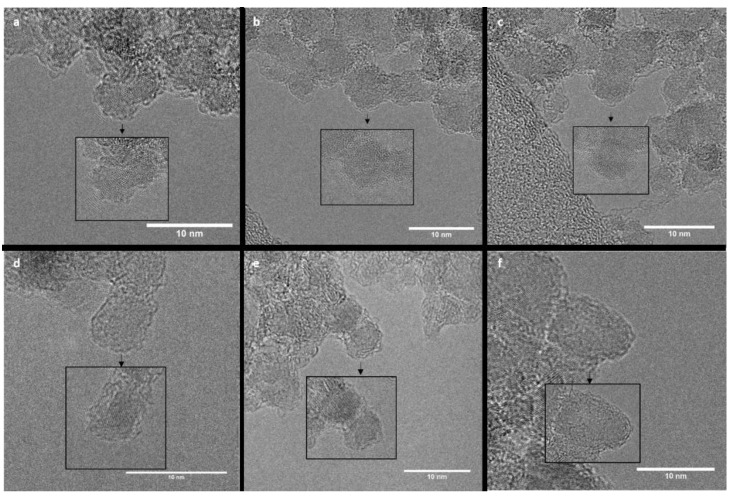
HR-TEM images of DND: (**a**–**c**) initial particles; (**d**) 800 °C 4 h; (**e**,**f**) 950 °C 4 h. Images recorded near to Scherzer defocus are provided into insets.

HR-TEM images were recorded to observe the impact of the annealing treatments on the DND morphology and crystalline structure (Figure 5). From the images, we can observe that the individual particles exhibit predominantly quasi-spherical shapes with diameters ranging from 2 nm to 8 nm, with a mean size of 6 nm. For the observed DND, different focalizations (Δf range between −70 nm to −90 nm) near the extended Scherzer defocus (|1.2 × ΔfScherzer| = 87.5 nm) were used on the same DND to maximize the phase contrast of a weak-phase object and resolution at the periphery of DND [51]. An example is presented in Figure 5d for a DND annealed at 800 °C under vacuum. Indeed, near the extended Scherzer defocus (inset), the diamond (111) planes are observed, extending to the particle borders. Among DND annealed at 950 °C under the same atmosphere, if a part is affected by phase contrast (Figure 5e), other DND exhibit modified outer shells (Figure 5f). According to HR-TEM investigations, the crystalline diamond core of DND is preserved, whatever the annealing temperature for both atmospheres. The observed modifications (Figure 5f) only affect the first outer shells for the highest temperature under vacuum.

## 4. Discussion

In the present study, chemical, structural, and morphological characterizations are combined to provide an accurate monitoring of the modifications underwent by detonation nanodiamonds during their annealing under a non-reactive atmosphere, i.e., vacuum or argon, from 800 °C up to 950 °C. Our aim was to reveal how the superficial chemistry of DND evolves at the early stages of graphitization and before their transformation into OLC, with particular attention given to the nature of the surface terminations and carbon hybridization. This work exhibited a noticeable difference between both atmospheres of annealing, which will be discussed below.

We started our experiments with particles presenting an oxidized surface, as evidenced by the prominent carboxyl band in FTIR and the XPS atomic percentage of oxygen reaching almost 10 at. %. In both conditions, from 800 °C, annealing induced a strong modification of the oxidized terminations, revealed by the XPS atomic percentage of oxygen brought down to roughly 5 at. % and the noticeable reduction and/or shift of the carbon-oxygen related IR absorption peaks. This remaining oxygen at the surface of our treated DND after annealing at 950 °C has to be discussed. Two options can be considered: (i) the impossibility to desorb certain kinds of carbon-oxygen groups even at 950 °C under vacuum or argon or (ii) a post-treatment spontaneous re-oxidation of the surface when exposed to air and/or dispersed in water. In the former case, persistence of carbon-oxygen functions above 800 °C will be only possible for carbonyl groups according to the literature [35,52]. This could explain the remaining of C=O stretching band at 1720 cm^−1^ visible on FTIR spectra of vacuum annealed samples. Note that this band vanishes while the annealing temperature is increasing. Concerning single-bounded carbon-oxygen groups revealed by FTIR and XPS C1s core levels, their presence would rather be linked to a spontaneous re-oxidation of the annealed DND surface when being in contact with air atmosphere/water. Spontaneous interactions between diamond surface and water molecules were probed by HREELS [53]. Depending on the diamond surface chemistry, these investigations reveal the formation of C–O and C=O bonds for a non-hydrogenated diamond. Such re-oxidation mechanism may also arise from the reactivity of sp^2^-C generated during annealing towards water molecules as suggested by XAS experiments [54]. This oxygen can also result from the reaction of ambient oxygen with defective graphitic structures generated during annealing on the DND surface [25].

Still concerning the surface terminations, our study emphasizes the creation of CH_x_ aliphatic bounds when DND are treated under vacuum or argon, as evidenced by infrared spectroscopy. Such CH_x_ are not detected by FTIR for initial DND in the same analysis conditions. It must be underlined here that IR spectroscopy probes the whole nanoparticle; therefore, we cannot discriminate if CH_x_ are created on or within the DND. Whatever its location, the origin of this hydrogen must be discussed. The creation of C–H bonds requires a hydrogen-rich atmosphere surrounding DND during the treatment or during the cooling down of the particles. We observed quite similar C–H stretching signatures (location and relative intensities) for both atmospheres, while they have been performed in dynamic primary vacuum at 10^−3^ mbar or under atmospheric pressure with a flow of argon at 20 sccm. Therefore, hydrogen coming from the degradation of desorbed functions (hydroxyls, carboxyl) and remaining in the vicinity of the particle is unlikely to be the only origin of the phenomenon. The hypothesis of hydrogen coming from the DND would appear more realistic, as the presence of such trapped hydrogen in the DND core was revealed previously by isotopic studies [55]. Indeed, hydrogen content in the range of 4 to 10 mg/g was measured by elemental analysis in DND of different origins [56] and shown to be kept inside the particle up to 750 °C [18]. The exodiffusion of hydrogen from the DND core was strengthened by a previous TDMS study performed on annealed DND. Hydrogen release was detected starting at 800 °C and raising up to 1000 °C [57]. To resume, we suggest here that the annealing of DND (at least from 800 °C) is accompanied by a thermodesorption of hydrogen initially trapped in the core, which induces the creation of CH_x_ functions through a catalytic pathway already described elsewhere [58].

Nevertheless, we observed a modification of C–H stretching bands according to the temperature treatment, which follows the same trend for primary vacuum and argon atmosphere. At 800 °C, the C–H stretching modes look similar to those obtained after a H_2_ annealing or plasma treatment [59], with two intense peaks centered at 2945 and 2879 cm^−1^. The nanodiamond literature reports two possibilities to attribute these peaks. Cheng and coworkers attributed these 2945 and 2879 cm^−1^ peaks to C(110):H and C(100):H, respectively, even on 5 nm detonation nanodiamonds [40]. In the meantime, several papers [60,61] since the pioneering one of Jiang et al. in 1996 [62] have described the doublet located at 2950 and 2871 cm^−1^ to the asymmetric and symmetric stretching vibrations of CH_3_ groups. Progressively, as the temperature of annealing raises, the 2922 and 2852 cm^−1^ bands become prominent, while the 2950 and 2871 cm^−1^ structures remain visible but less intense. This would mean a transition between CH_3_ to CH_2_ groups according to Jiang’s papers, which should be driven by the annealing temperature and the concomitant reconstruction of the nanodiamond surface while isolated graphitic structures cover the particle. On the other hand, this evolution may also be due to an evolution of mean diameter of the particles probed, towards smaller sizes, if we consider the work of Cheng and coworkers. However, considering our HR-TEM observations, the diameters of annealed DND do not seem to be reduced, which rules out this hypothesis.

The infrared analysis puts also into light the apparition of a large band ranging from 1620 to 1550 cm^−1^ and centered around 1580 cm^−1^ with the annealing procedure, whatever the atmosphere. A part of this area (1620–1590 cm^−1^) can be attributed to red-shifted OH bending modes related to specific water interaction with hydrophobic surface, which could be the case here, as our DND surface seems to become hydrogenated and may exhibit some graphitic carbon at its surface [39,63]. However, this specific OH bending mode should be accompanied with an OH stretching mode between 3620 and 3690 cm^−1^ also specific to hydrophobic surfaces [38], which is not visible in our spectra. Furthermore, to check if adsorbed water could be responsible for that band, annealed DND were suspended in D_2_O; this band was still detected (Figure S2) despite the expected isotopic shift of OD band. Therefore, despite hydrogen and sp^2^ carbon on their surface, our treated DND do not seem to exhibit clear hydrophobic behavior. Still in the same area, another contribution may concern vinyl C=C bounds (around 1580 cm^−1^) [64,65]. However, if this band is related to C=C bonds, and by extension to graphitic reconstructions, XPS data does not support the infrared analysis since this band at 1580 cm^−1^ is prominent for samples annealed under argon and weaker for those annealed under vacuum, while higher sp^2^-C content was evidenced on C1s core levels of vacuum annealed samples. In the meantime, quantification on infrared absorption spectrum remains hazardous.

Thermal treatments are expected to lead to the creation of graphitic carbon on DND. In this study, Raman spectroscopy was initially used to reveal this evolution of the sp^3^ carbon to sp^2^ carbon. Here, what is shown by Raman spectroscopy is mainly the loss of the first order diamond peak which is hidden by the D band, most related to disordered carbon. However, HR-TEM images performed on the same samples confirm that the diamond core is preserved, even for the highest temperature. Indeed, at this excitation wavelength of 532 nm, the scattering of “graphitic” carbons (D and G bands) is exalted compared to diamond, by a factor of 50 [43]. This is a good illustration of the difficulty to use such green excitation to probe nanodiamond structure by Raman spectroscopy, especially when annealing treatments are considered [42].

XPS analysis provides a quantitative study of the graphitization. From 3.5% of the overall carbon probed on initial DND, this proportion of sp^2^-hybridized carbon went up to 15 and 30% for annealing performed under argon and vacuum, respectively. We observe with this technique the first noticeable difference induced by the atmosphere. Note that annealing temperatures over the experimental range (800–950 °C) were checked in situ using an additional thermocouple, and comparable values were measured, whatever the atmosphere. In our study, vacuum annealing definitely induces more sp^2^-hybridized carbon in this temperature range than the same treatment realized at atmospheric pressure under an inert atmosphere. The cause of this observation is most probably related to the reactivity of the carbon dangling bonds at low pressure. Now, if we look deeper in the values of created sp^2^-hybridized carbon provided by XPS, the maximum of 30% may suggest a thick graphic layer surrounding DND. However, HR-TEM images do not evidence such structures, revealing only very peripheral modifications of the DND, even at 950 °C. This can be explained by a “nano” effect that should be taken into consideration in our XPS analysis. Due to geometrical consideration and when the radius of the particle approaches the inelastic mean free path of electrons (≈2 nm in diamond [66]), well described by Baer and Engelhard [67,68], a 2.5-fold enhancement of the carbon signal coming from the first 0.5 nm of a ≈5 nm nanoparticle occurs. Therefore, in our case, if we consider that sp^2^ carbon is located at the extreme surface of DND as seen in HR-TEM, this ratio of 30% can be reduced to 12%, which roughly represents one layer of carbon atoms at the surface of a ≈5 nm nanoparticle. The thickness of sp^2^ layer estimated from HR-TEM images is included between 0.45 and 0.58 nm. This corrected amount of sp^2^-hybridized carbon is more in agreement with our HR-TEM observations, considering that for a ≈ 5 nm diamond particle, 15% of carbon atoms are located at the surface [69]. This observation was confirmed by an additional thermal treatment we performed under vacuum at 1100 °C. On this sample, HR-TEM clearly exhibits the formation of more organized graphitic structures at the extreme surface of DND (Figure S3), but still limited to a monolayer. In agreement, sp^2^-hybridized carbon component of the C1s core level spectrum remains around 30% (Figure S4). However, we noticed a down-shift of the sp^2^-hybridized carbon component with respect to the sp^3^ C–C diamond up to −1.3 eV. This down-shift may reflect the higher degree of organization of this graphitic structure as seen on HR-TEM images. Note that this exaltation of the sp^2^-hybridized carbon also occurs for initial and low-temperature annealed DND. This means that less than 1% of sp^2^-hybridized is present on initial particles, in full agreement with HR-TEM images.

XPS analysis provides complementary information about the crystalline structure of treated nanodiamonds. Initially, the component related to sp^3^ C–C neighboring structural defects, carbon partially saturated with hydrogen and carbon bounded to a nitrogen atom counts for more than half of the C1s total area (53%). At the same time, the component relative to the sp^3^ C–C diamond type only represents 6% of the total carbon probed. After annealing at 800 or 850°C, according to the atmosphere, a reorganization of the C–C sp^3^ bonds seems to occur, as the latter component grows up to more than 40% of the C1s core level, while the former is notably reduced. It is well known in the field of NV (nitrogen vacancy) center synthesis that annealing above 750 °C leads to a migration of vacancies contained in the nanodiamond core to meet nitrogen atoms to form NV centers, while some vacancies are released to the surface [50]. The raise of the sp^3^ C–C diamond type component from 6 to 41% may be explained by this effect, i.e., due to the “healing” of the core defects. Such an effect was evidenced by electron paramagnetic resonance (EPR) with a reduced density of spins, by a factor of two, for DND annealed at 600 °C compared to initial particles [34]. This is assigned to a reduction of dangling bonds in the sp^3^ diamond core. At the same time, the second component initially linked to these defects is progressively replaced by the reconstructed surface, left by the desorption of the oxygen-related groups at the surface of the particle. Note that part of these evolutions may be exalted by the “nano effect” as explained above for superficial carbon bounds. In our experiments, we also noticed that sp^3^ C–C diamond component, related to the core of the DND, is reduced from 45 to 29% when high temperatures are reached, partly replaced by sp^2^-hybridized carbon component and sp^3^ C–C neighboring structural defects. In this temperature range, some carbon atoms from the diamond core underwent structural modifications, leading to more defects in the diamond lattice. The HR-TEM images of the additional experiments at 1100 °C in vacuum (Figure S3) illustrate this hybrid structure with a diamond core surrounded with defective layers and covered with a more organized sp^2^ layer, exhibiting a cubic symmetry as shown by the FFT presented on Figure S5).

## 5. Conclusions

An accurate control of the early stages of DND graphitization is desirable to further exploit the outstanding properties of the nanodiamonds in the fields of energy science and nanomedicine [16], or towards efficient functionalization [18]. In the present work, DND from Adamas Nanotechnology were annealed in the 800–950 °C range under two atmospheres in the same set-up: vacuum at 10^−3^ mbar or argon flow at atmospheric pressure. The effects of thermal treatments on DND surface chemistry, crystallinity, and carbon hybridization were investigated via complementary techniques (FTIR, Raman, XPS, and HR-TEM).

In the 800–950 °C range, a very progressive formation of sp^2^-hybridized carbon was evidenced and quantified by XPS and appeared to be more efficient for DND annealed under vacuum (up to 30% of the total carbon) than under argon at atmospheric pressure. Taking into account the nano-effect reported in XPS for 5–6 nm particles [69], the highest sp^2^ amount obtained in this study corresponds to a monolayer, in fair agreement with the HR-TEM images. Therefore, by adjusting the temperature within this range of temperatures, we showed that it is possible to finely tune the DND surface chemistry into a hybrid structure presenting sp^2^ and sp^3^ carbons at its surface. Note that pushing the treatment up to 1100°C under vacuum does not increase the amount of sp^2^-hybridized carbon but gives rise to a better-organized aromatic structure. We also evidenced by XPS the “self-healing” of the defects initially present in the DND cores when they are exposed to 800 °C (or above). Another point of high interest revealed here is the systematic appearance of C–H stretching bands in all annealed samples, whatever the atmosphere. The exodiffusion of hydrogen from the diamond core and the subsequent creation of aliphatic C–H bonds are the most probable origins of this effect.

In summary, our study provides a fine characterization of the early stages of graphitization of DND. Our results emphasize that within the 800–1100 °C range and for this source of nanodiamonds, it is possible to finely control the amount and the crystallinity of sp^2^-hybridized carbon at the periphery of DND mainly from the first outer-shell, while the coexistence of C–H functions cannot be avoided. It may be of interest to investigate these early stages of graphitization on other DND sources to better define how these results are particle dependent.

## Figures and Tables

**Table 1:** Oxygen and nitrogen atomic concentrations determined by XPS for initial and annealed DND for both atmospheres. The uncertainty on concentration is estimated to ±0.5 at. %.

	Temperature °C	Oxygen at. %	Nitrogen at. %
**Initial**		9.3	1.9
**Vacuum**	800	5.7	1.6
	850	5.3	1.3
	900	6.1	1.2
	950	5.2	1.3
**Argon**	800	5.1	1.5
	850	3.6	1.5
	900	5.0	1.4
	950	4.0	1.3

## Supplementary Materials

The following are available online at https://www.mdpi.com/article/10.3390/nano11102671/s1, Figure S1: Fitted XPS C1s core levels and associated values, Figure S2: FTIR of DND annealed at 800 °C suspended in D_2_O, Figure S3: HR-TEM images of DND annealed at 1100 °C under vacuum, Figure S4: Fitted XPS C1s core level of DNDs annealed at 1100 °C under vacuum and associated values, Figure S5: HR-TEM images and FFT for as received DND and DND annealed under vacuum at 950 °C and 1100 °C.

## References

[1] Turcheniuk K., Mochalin V.N. (2017). Biomedical applications of nanodiamond (Review). Nanotechnology.

[2] Navalón S., Dhakshinamoorthy A., Álvaro M., García H. (2020). Diamond Nanoparticles in Heterogeneous Catalysis. Chem. Mater..

[3] Ivanov M., Shenderova O. (2017). Nanodiamond-based nanolubricants for motor oils. Curr. Opin. Solid State Mater. Sci..

[4] Zhang Y., Rhee K.Y., Hui D., Park S.J. (2018). A critical review of nanodiamond based nanocomposites: Synthesis, properties and applications. Compos. Part. B Eng..

[5] Wang H., Lee D.-K., Chen K.-Y., Chen J.-Y., Zhang K., Silva A., Ho C.-M., Ho D. (2015). Mechanism-Independent Optimization of Combinatorial Nanodiamond and Unmodified Drug Delivery Using a Phenotypically Driven Platform Technology. ACS Nano.

[6] Van der Laan K., Hasani M., Zheng T., Schirhagl R. (2018). Nanodiamonds for In Vivo Applications. Small.

[7] Choi H.S., Liu W., Misra P., Tanaka E., Zimmer J.P., Itty Ipe B., Bawendi M.G., Frangioni J.V. (2007). Renal clearance of quantum dots. Nat. Biotechnol..

[8] Claveau S., Nehlig É., Garcia-Argote S., Feuillastre S., Pieters G., Girard H.A., Arnault J.-C., Treussart F., Bertrand J.-R. (2020). Delivery of siRNA to Ewing Sarcoma Tumor Xenografted on Mice, Using Hydrogenated Detonation Nanodiamonds: Treatment Efficacy and Tissue Distribution. Nanomaterials.

[9] Shvidchenko A.V., Eidelman E.D., Vul’ A.Y., Kuznetsov N.M., Stolyarova D.Y., Belousov S.I., Chvalun S.N. (2019). Colloids of detonation nanodiamond particles for advanced applications. Adv. Colloid Interface Sci..

[10] Mironova E.Y., Ermilova M.M., Efimov M.N., Zemtsov L.M., Orekhova N.V., Karpacheva G.P., Bondarenko G.N., Zhilyaeva N.A., Muraviev D.N., Yaroslavtsev A.B. (2013). Detonation nanodiamonds as catalysts of steam reforming of ethanol. Russ. Chem. Bull..

[11] Zhang L., Hamers R.J. (2017). Photocatalytic reduction of CO_2_ to CO by diamond nanoparticles. Diam. Relat. Mater..

[12] Mi Z., Chen C.-B., Tan H.Q., Dou Y., Yang C., Turaga S.P., Ren M., Vajandar S.K., Yuen G.H., Osipowicz T. (2021). Quantifying nanodiamonds biodistribution in whole cells with correlative iono-nanoscopy. Nat. Commun..

[13] Grall R., Girard H., Saad L., Petit T., Gesset C., Combis-Schlumberger M., Paget V., Delic J., Arnault J.-C., Chevillard S. (2015). Impairing the radioresistance of cancer cells by hydrogenated nanodiamonds. Biomaterials.

[14] Brun E., Girard H.A., Arnault J., Mermoux M., Sicard-Roselli C. (2020). Hydrogen plasma treated nanodiamonds lead to an overproduction of hydroxyl radicals and solvated electrons in solution under ionizing radiation. Carbon N. Y..

[15] Wehling J., Dringen R., Zare R.N., Maas M., Rezwan K. (2014). Bactericidal activity of partially oxidized nanodiamonds. ACS Nano.

[16] Lin Y., Sun X., Su D.S., Centi G., Perathoner S. (2018). Catalysis by hybrid sp^2^/sp^3^ nanodiamonds and their role in the design of advanced nanocarbon materials. Chem. Soc. Rev..

[17] Yang K., Wan J., Zhang S., Tian B., Zhang Y., Liu Z. (2012). The influence of surface chemistry and size of nanoscale graphene oxide on photothermal therapy of cancer using ultra-low laser power. Biomaterials.

[18] Liang Y., Meinhardt T., Jarre G., Ozawa M., Vrdoljak P., Schöll A., Reinert F., Krueger A. (2011). Deagglomeration and surface modification of thermally annealed nanoscale diamond. J. Colloid Interface Sci..

[19] Su D., Maksimova N.I., Mestl G., Kuznetsov V.L., Keller V., Schlögl R., Keller N. (2007). Oxidative dehydrogenation of ethylbenzene to styrene over ultra-dispersed diamond and onion-like carbon. Carbon N. Y..

[20] Kuznetsov V.L., Butenko Y.V., Shenderova O.A., Gruen D.M. (2012). Diamond phase transitions at nanoscale. Ultananocrystalline Diamond.

[21] Zeiger M., Jäckel N., Mochalin V.N., Presser V. (2016). Review: Carbon onions for electrochemical energy storage. J. Mater. Chem. A.

[22] Mykhaylyk O.O., Solonin Y.M., Batchelder D.N., Brydson R. (2005). Transformation of nanodiamond into carbon onions: A comparative study by high-resolution transmission electron microscopy, electron energy-loss spectroscopy, X-ray diffraction, small-angle X-ray scattering, and ultraviolet Raman spectroscopy. J. Appl. Phys..

[23] Zeiger M., Jäckel N., Aslan M., Weingarth D., Presser V. (2015). Understanding structure and porosity of nanodiamond-derived carbon onions. Carbon N. Y..

[24] Plonska-Brzezinska M.E., Molina-Ontoria A., Echegoyen L. (2014). Post-modification by low-temperature annealing of carbon nano-onions in the presence of carbohydrates. Carbon N. Y..

[25] Xie F.Y., Xie W.G., Gong L., Zhang W.H., Chen S.H., Zhang Q.Z., Chen J. (2010). Surface characterization on graphitization of nanodiamond powder annealed in nitrogen ambient. Surf. Interface Anal..

[26] Aleksenskii A.E., Baidakova M.V., Vul A.Y., Dideikin A.T., Siklitskii V.I., Vul’ S.P. (2000). Effect of hydrogen on the structure of ultradisperse diamond. Phys. Solid State.

[27] Fugaciu F., Hermann H., Seifert G. (1999). Concentric-shell fullerenes and diamond particles: A molecular-dynamics study. Phys. Rev. B.

[28] Kuznetsov V.L., Zilberberg I.L., Butenko Y.V., Chuvilin A.L., Segall B. (1999). Theoretical study of the formation of closed curved graphite-like structures during annealing of diamond surface. J. Appl. Phys..

[29] Raty J.-Y., Galli G., Bostedt C., van Buuren T., Terminello L. (2003). Quantum Confinement and Fullerenelike Surface Reconstructions in Nanodiamonds. Phys. Rev. Lett..

[30] Ray M.A., Shenderova O., Hook W., Martin A., Grishko V., Tyler T., Cunningham G.B., McGuire G. (2006). Cold plasma functionalization of nanodiamond particles. Diam. Relat. Mater..

[31] Petit T., Arnault J.C., Girard H.A., Sennour M., Bergonzo P. (2011). Early stages of surface graphitization on nanodiamond probed by X-ray photoelectron spectroscopy. Phys. Rev. B Condens. Matter Mater. Phys..

[32] Petit T., Arnault J.-C., Girard H.A., Sennour M., Kang T.-Y., Cheng C.-L., Bergonzo P. (2012). Oxygen hole doping of nanodiamond. Nanoscale.

[33] Cebik J., McDonough J.K., Peerally F., Medrano R., Neitzel I., Gogotsi Y., Osswald S. (2013). Raman spectroscopy study of the nanodiamond-to-carbon onion transformation. Nanotechnology.

[34] Panich A.M., Shames A.I., Sergeev N.A., Olszewski M., McDonough J.K., Mochalin V.N., Gogotsi Y. (2013). Nanodiamond graphitization: A magnetic resonance study. J. Phys. Condens. Matter.

[35] Koshcheev A.P. (2009). Thermodesorption mass spectrometry in the light of solution of the problem of certification and unification of the surface properties of detonation nano-diamonds. Russ. J. Gen. Chem..

[36] Petit T., Puskar L. (2018). FTIR spectroscopy of nanodiamonds: Methods and interpretation. Diam. Relat. Mater..

[37] Ackermann J., Krueger A. (2019). Efficient surface functionalization of detonation nanodiamond using ozone under ambient conditions. Nanoscale.

[38] Stehlik S., Glatzel T., Pichot V., Pawlak R., Meyer E., Spitzer D., Rezek B. (2016). Water interaction with hydrogenated and oxidized detonation nanodiamonds—Microscopic and spectroscopic analyses. Diam. Relat. Mater..

[39] Petit T., Puskar L., Dolenko T., Choudhury S., Ritter E., Burikov S., Laptinskiy K., Brzustowski Q., Schade U., Yuzawa H. (2017). Unusual Water Hydrogen Bond Network around Hydrogenated Nanodiamonds. J. Phys. Chem. C.

[40] Cheng C.-L., Chen C.-F., Shaio W.-C., Tsai D.-S., Chen K.-H. (2005). The CH stretching features on diamonds of different origins. Diam. Relat. Mater..

[41] Aleksenskiǐ A.E., Baǐdakova M.V., Vul A.Y., Davydov V.Y., Pevtsova Y.A. (1997). Diamond-graphite phase transition in ultradisperse-diamond clusters. Phys. Solid State.

[42] Mermoux M., Crisci A., Petit T., Girard H.A., Arnault J.-C. (2014). Surface Modifications of Detonation Nanodiamonds Probed by Multiwavelength Raman Spectroscopy. J. Phys. Chem. C.

[43] Knight D.S., White W.B. (1989). Characterization of diamond films by Raman spectroscopy. J. Mater. Res..

[44] Brenier R., Mugnier J., Mirica E. (1999). XPS study of amorphous zirconium oxide films prepared by sol-gel. Appl. Surf. Sci..

[45] Ozawa M., Inaguma M., Takahashi M., Kataoka F., Krüger A., Ōsawa E. (2007). Preparation and Behavior of Brownish, Clear Nanodiamond Colloids. Adv. Mater..

[46] Dolmatov V.Y. (2009). On elemental composition and crystal-chemical parameters of detonation nanodiamonds. J. Superhard Mater..

[47] Arnault J.C. (2018). X-ray Photoemission Spectroscopy applied to nanodiamonds: From surface chemistry to in situ reactivity. Diam. Relat. Mater..

[48] Stehlik S., Mermoux M., Schummer B., Vanek O., Kolarova K., Stenclova P., Vlk A., Ledinsky M., Pfeifer R., Romanyuk O. (2021). Size effects on surface chemistry and Raman spectra of Sub-5 nm oxidized high-pressure high-temperature and detonation nanodiamonds. J. Phys. Chem. C.

[49] Girard H., Simon N., Ballutaud D., Herlem M., Etcheberry A. (2007). Effect of anodic and cathodic treatments on the charge transfer of boron doped diamond electrodes. Diam. Relat. Mater..

[50] Smith J.M., Meynell S.A., Bleszynski Jayich A.C., Meijer J. (2019). Colour centre generation in diamond for quantum technologies. Nanophotonics.

[51] Turner S., Lebedev O.I., Shenderova O., Vlasov I.I., Verbeeck J., Van Tendeloo G. (2009). Determination of Size, Morphology, and Nitrogen Impurity Location in Treated Detonation Nanodiamond by Transmission Electron Microscopy. Adv. Funct. Mater..

[52] Figueiredo J.L., Pereira M.F.R., Freitas M.M.A., Órfão J.J.M. (1999). Modification of the surface chemistry of activated carbons. Carbon N. Y..

[53] Laikhtman A., Lafosse A., Le Coat Y., Azria R., Hoffman A. (2004). Interaction of water vapor with bare and hydrogenated diamond film surfaces. Surf. Sci..

[54] Petit T., Pflüger M., Tolksdorf D., Xiao J., Aziz E.F. (2015). Valence holes observed in nanodiamonds dispersed in water. Nanoscale.

[55] Nehlig E., Garcia-Argote S., Feuillastre S., Moskura M., Charpentier T., Schleguel M., Girard H.A., Arnault J.-C., Pieters G. (2019). Using hydrogen isotope incorporation as a tool to unravel the surfaces of hydrogen-treated nanodiamonds. Nanoscale.

[56] Mitev D.P., Townsend A.T., Paull B., Nesterenko P.N. (2014). Microwave-assisted purification of detonation nanodiamond. Diam. Relat. Mater..

[57] Arnault J.C., Petit T., Girard H.A., Gesset C., Combis-Schlumberger M., Sennour M., Koscheev A., Khomich A.A., Vlasov I., Shenderova O. (2014). Surface graphitization of ozone treated detonation nanodiamonds. Phys. Status Solidi Appl. Mater. Sci..

[58] Ahmed A.-I., Mandal S., Gines L., Williams O.A., Cheng C.-L. (2016). Low temperature catalytic reactivity of nanodiamond in molecular hydrogen. Carbon N. Y..

[59] Thalassinos G., Stacey A., Dontschuk N., Murdoch B.J., Mayes E., Girard H.A., Abdullahi I.M., Thomsen L., Tadich A., Arnault J. (2020). Fluorescence and Physico-Chemical Properties of Hydrogenated Detonation Nanodiamonds. C J. Carbon Res..

[60] Shenderova O., Panich A.M., Moseenkov S., Hens S.C., Kuznetsov V., Vieth H.-M. (2011). Hydroxylated Detonation Nanodiamond: FTIR, XPS, and NMR Studies. J. Phys. Chem. C.

[61] Williams O.A., Hees J., Dieker C., Jäger W., Kirste L., Nebel C.E. (2010). Size-Dependent Reactivity of Diamond Nanoparticles. ACS Nano.

[62] Jiang T., Xu K., Ji S., Jib S. (1996). FTIR studies on the spectral changes of the surface functional groups of ultradispersed diamond powder synthesized by explosive detonation after treatment in hydrogen, nitrogen, methane and air at different temperatures. J. Chem. Soc. Faraday Trans..

[63] Stehlik S., Henych J., Stenclova P., Kral R., Zemenova P., Pangrac J., Vanek O., Kromka A., Rezek B. (2021). Size and nitrogen inhomogeneity in detonation and laser synthesized primary nanodiamond particles revealed via salt-assisted deaggregation. Carbon.

[64] Scholz J., McQuillan A.J., Holt K.B. (2011). Redox transformations at nanodiamond surfaces revealed by in situ infrared spectroscopy. Chem. Commun..

[65] Liang Y., Ozawa M., Krueger A. (2009). A General Procedure to Functionalize Agglomerating Nanoparticles Demonstrated on Nanodiamond. ACS Nano.

[66] Tanuma S., Powell C.J., Penn D.R. (2011). Calculations of electron inelastic mean free paths. IX. Data for 41 elemental solids over the 50 eV to 30 keV range. Surf. Interface Anal..

[67] Baer D., Engelhard M., Gaspar D. (2005). Challenges in Applying Surface Analysis Methods to Nanoparticles and Nanostructured Materials. J. Surf. Anal..

[68] Baer D.R., Engelhard M.H. (2010). XPS analysis of nanostructured materials and biological surfaces. J. Electron. Spectros. Relat. Phenom..

[69] Shenderova O.A., McGuire G.E. (2015). Science and engineering of nanodiamond particle surfaces for biological applications (Review). Biointerphases.

