# Supplementary material for: New Insights into the Reactivity of Detonation Nanodiamonds during the First Stages of Graphitization
Source: Nanomaterials (Basel). 2021 Oct 11;11(10):2671. doi: 10.3390/nano11102671 (PMC8537936; doi:10.3390/nano11102671)
Supplement: Supplementary file 1 [file nanomaterials-11-02671-s001.zip › nanomaterials-1413118-supplementary.pdf]

nanomaterials

MDPI

Supplementary Materials

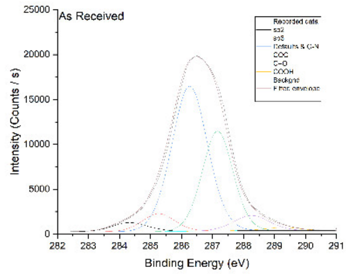

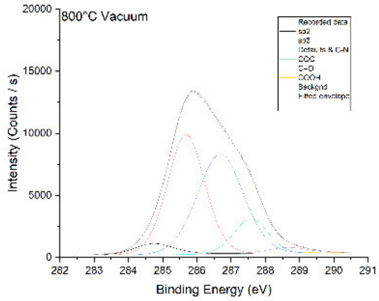

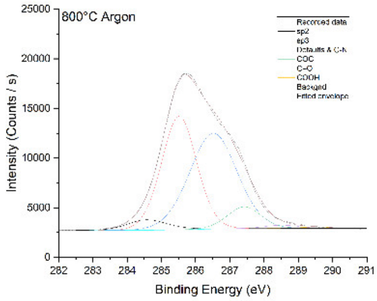

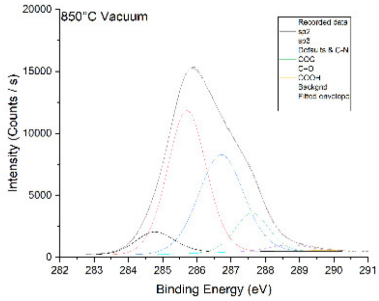

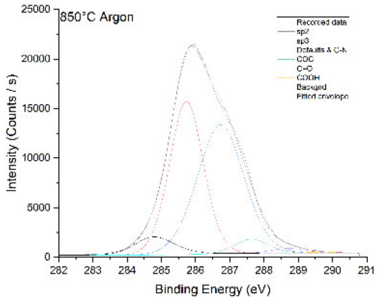

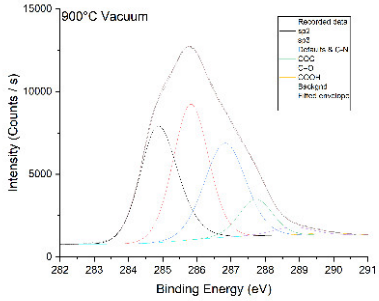

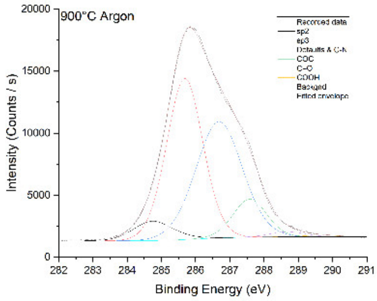

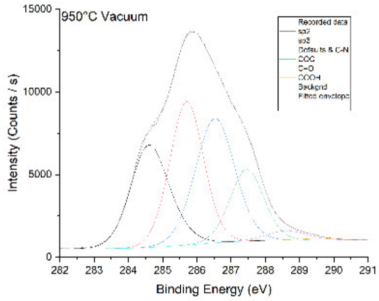

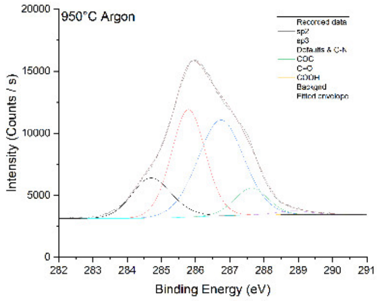

Argon annealing						
	C sp^2	C sp^3	CHx, C-N, defaults	C-O-C	C=O	COOH
As received	3,5	6,0	53,0	31,0	5,5	1,0
800°C 4h	4,5	40,5	46,0	7,5	1,0	0,5
850°C 4h	6,0	41,0	47,5	4,0	1,0	0,5
900°C 4h	6,0	43,0	40,0	9,5	1,0	0,5
950°C 4h	15,0	34,0	41,0	8,5	1,0	0,5

Vacuum annealing						
	C sp^2	C sp^3	CHx, C-N, defaults	C-O-C	C=O	COOH
As received	3,5	6	53	31	5,5	1
800°C 4h	4,5	41	41,5	10,5	2	0,5
850°C 4h	7,5	45	35	10	2	0,5
900°C 4h	30	32,5	26,5	8,5	2	0,5
950°C 4h	24	29	30	14,5	2	0,5

Figure S1. Fitted XPS C1s core levels and associated values.

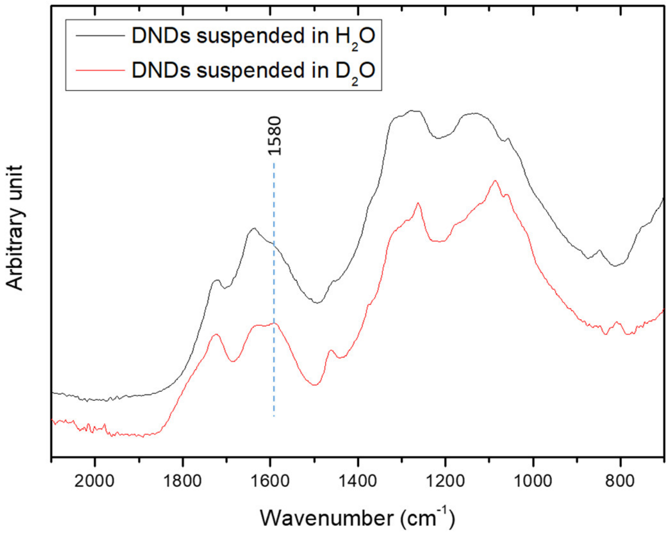

Figure S2. FTIR of DNDs annealed at 800 °C for 4 h suspended in H_2O and D_2O.

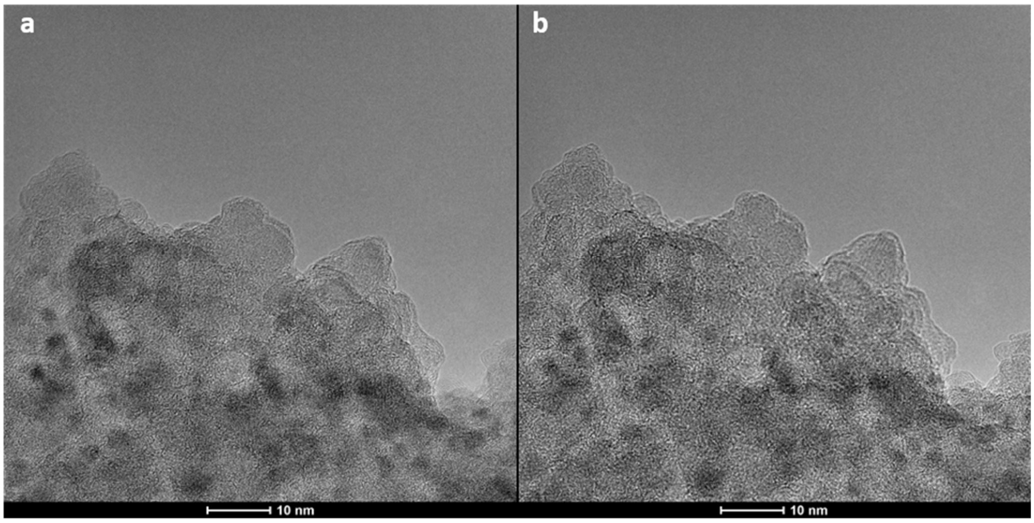

Figure S3. HR-TEM images with different defocus values of DNDs annealed at 1100 °C under vacuum.

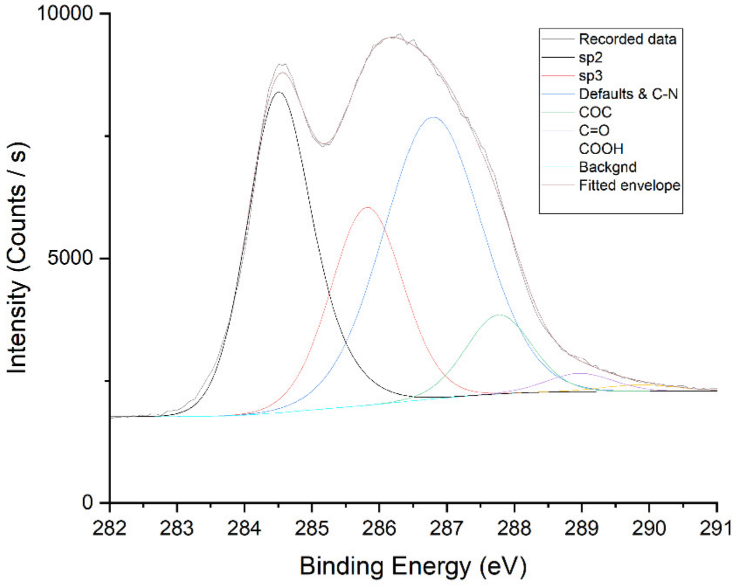

Vacuum annealing						
	C sp^2	C sp^3	CHx, C-N, defaults	C-O-C	C=O	COOH
1100°C 4h	30,5	20	39,5	7,5	2	0,5

Figure S4. Fitted XPS C1s core level of DNDs annealed at 1100 °C under vacuum and associated values.

As received DND

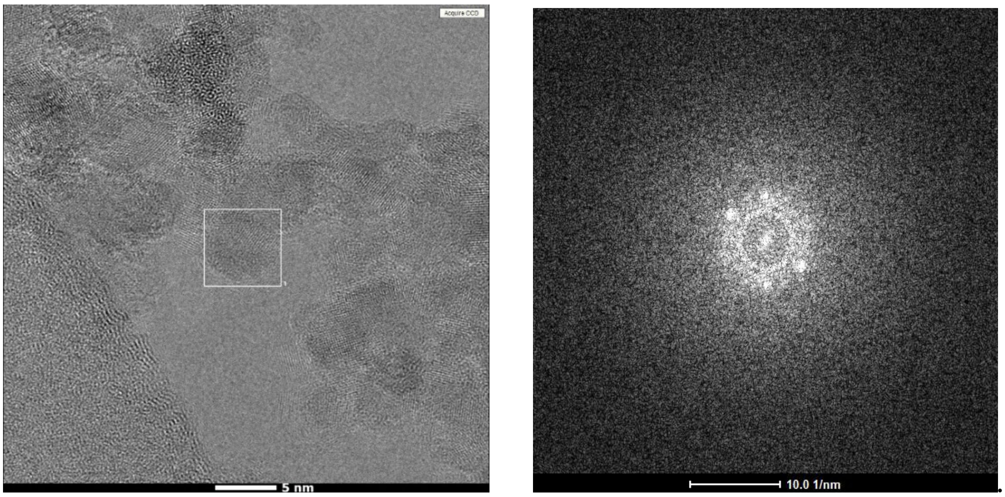

DND annealed under vacuum at 950 °C

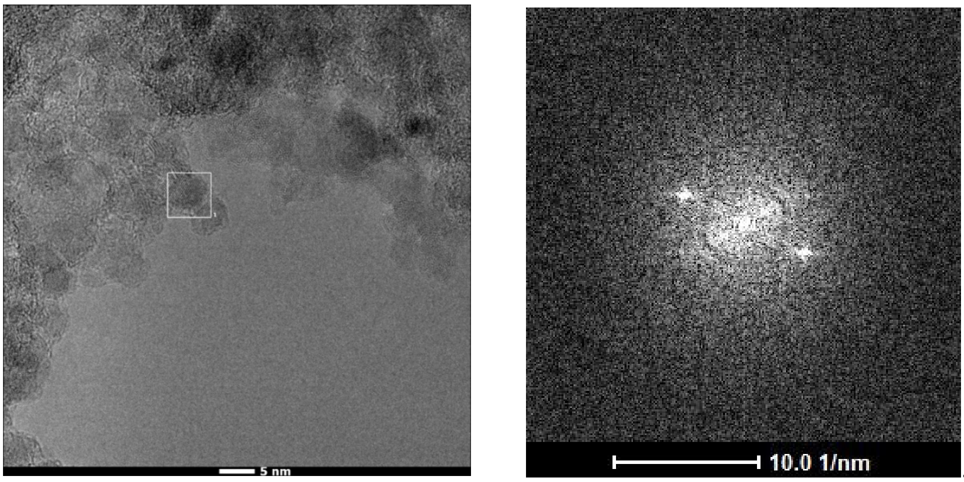

DND annealed under vacuum at 1100 °C

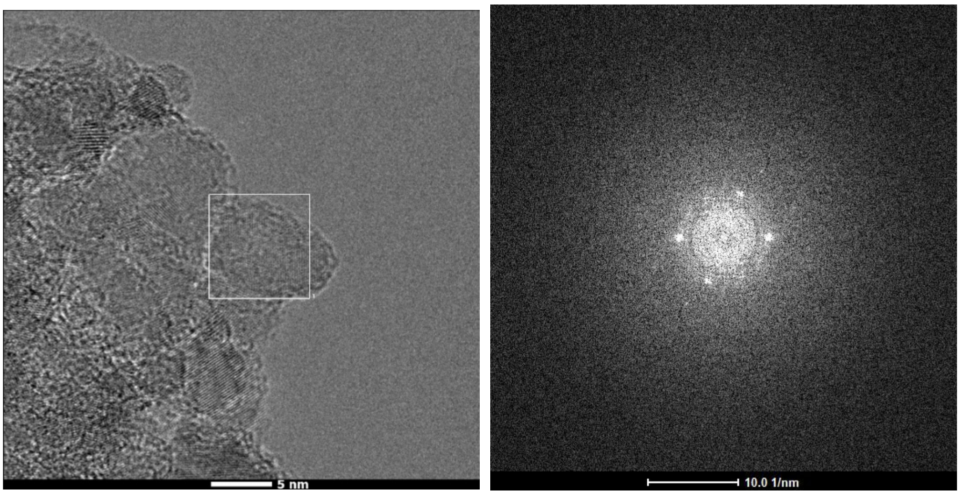

Figure S5. HR-TEM images (left) and FFT (right) performed in the area corresponding to the red square.
